# OPN5 Regulating Mechanism of Follicle Development Through the TSH-DIO2/DIO3 Pathway in Mountain Ducks Under Different Photoperiods

**DOI:** 10.3389/fphys.2022.813881

**Published:** 2022-06-01

**Authors:** Sui Liufu, Jianqiu Pan, Junfeng Sun, Xu Shen, Danli Jiang, Hongjia Ouyang, Danning Xu, Yunbo Tian, Yunmao Huang

**Affiliations:** ^1^ College of Animal Science & Technology, Zhongkai University of Agriculture and Engineering, Guangzhou, China; ^2^ Guangdong Province Key Laboratory of Waterfowl Healthy Breeding, Guangzhou, China

**Keywords:** Opn5, TSH-DIO2/DIO3 pathway, photoperiod, Follicular development, mountain duck

## Abstract

**Abstract:** Photoperiod is an important environmental factor that influence seasonal reproduction behavior in bird. Birds translates photoperiodic information into neuroendocrine signals through deep brain photoreceptors (DBPs). OPN5 has been considered as candidate DBPs involving in regulation of seasonal reproduction in birds. However, little is known about the effect of OPN5 in non-seasonal breeding birds. Thus, we pondered on whether OPN5 regulating follicular development through TSH-DIO2/DIO3 system responds to different photoperiods in non-seasonal laying ducks. As an ideal non-seasonal breeding bird, a total of 120 mountain ducks were randomly divided into three groups and treated respectively to a different photoperiod: group S (8 L:16D), group C (17 L:7D), and group L (24 L:0D). The ducks were caged in a fully enclosed shelter with the same feeding conditions for each group, free water and limited feeding (150 g per duck each day). Samples were collected from each group at d 0, d 5, d 8, d 20, and d 35 (*n* = 8). The ducks in 24 h photoperiod had the highest laying rate and the lowest feed-to-egg ratio, while the ducks in 8 h photoperiod had the lowest laying rate and the highest feed-to-egg ratio. Long-day photoperiod for 24 h significantly increased the ovarian index and GnRH, LH, E2, and P4 levels in serum; short-day photoperiod for 8 h increased testosterone levels in serum. Compared with 8 h photoperiod, long-day photoperiod significantly or highly significantly increased the mRNA level and protein expression of OPN5 in the hypothalamus of long-day photoperiod on d 35 (*p* < 0.05). The gene or protein expression patterns of GnRH, TRH, TSHβ, DIO2, THRβ, VIP, and PRL were positively correlated with OPN5, whereas the gene expression patterns of *GnIH* and *DI O 3* were negatively correlated with *OPN5*. The results revealed that OPN5 mediated the effect of light on follicular development through the TSH-DIO2/DIO3 pathway, the expression of OPN5 increased with light duration and improved the efficiency of the HPG axis to promote follicular development in mountain ducks.

## 1 Introduction

Opsin 5(OPN5) is a novel G protein-coupled receptor (GPCR), firstly identified in mammalian neural tissue in 2003 (Tarttelin et al., 2003; Nakane and Yoshimua, 2019), and mainly expressed in the retina, hypothalamic paraventricular organ (PVO), and gonads (Kojima et al., 2011; Stevenson and Ball, 2012). OPN5 is the main deep-brain photoreceptor (DBP) in birds (Nakane and Yoshimua, 2019), which is involved in regulating reproductive functions (Volle, 2021). Three main DBPs have been identified, including melanopsin (OPN4), neurophotoprotein (OPN5), and vertebra ancient opsin (VAOpn) (Kang and Kuenzel, 2015; Beaudry et al., 2017). OPN5 is a short-wavelength sensitive photopigment that absorbs at 360–474 nm and is thought to be a UV-sensitive serine protease capable of mediating light signaling (Kojima et al., 2011). It has been confirmed that OPN5 can affect bird reproduction through the hypothalamic-pituitary-gonadal axis (HPG axis) (Zhu et al., 2019a; Zhu et al., 2019b). A large number of studies (Kuenzel et al., 2015; Mishra et al., 2017; Mishra et al., 2018) have shown that OPN5 in the paraventricular nucleus of birds could transmit light signals to the pituitary nodule (PT) in order to initiate the thyroid hormone response (TH-responsive) signaling pathway, which has effects on the production and secretion of Gonadotropin-releasing hormone (GnRH) by regulating the secretion of triiodothyronine (T3) (Saelim et al., 2004). Therefore, OPN5 plays an important role in the light regulation of bird reproductive activities.

Reproduction in birds is regulated by the hypothalamic-pituitary-gonadal axis, and the HPG axis is a complete feedback system formed by the hypothalamus, pituitary, and gonads under central nervous regulation (Rose et al., 2022). Hypothalamus secretes GnRH and Gonadotropin-inhibitory hormone (GnIH) to regulate gonadotropin secretion at the pituitary level (Nabi et al., 2020). Gonadotropins regulate the secretion of gonadotropic hormones through blood circulation, which in turn promotes gonadal development and regulates animal reproduction (Zhu et al., 2019a; Zhu et al., 2019b). In addition, in seasonally breeding avian species, nesting behavior associated with reproduction is an important biological feature and is related to neurotransmitters produced by the hypothalamus, pituitary gland and hypothalamus-pituitary-Gonadal axis (HPG)reproductive hormones (Saelim et al., 2004; Gumułka et al., 2020). The HPG axis secretes a variety of hormones, including GnRH hormone at the hypothalamic level, prolactin (PRL),follicle-stimulating hormone (FSH), and luteinizing hormone (LH) hormones at the pituitary level, and E2 and P4 at the gonadal level and the nesting behavior is mediated by the action of ovarian E2 on the hypothalamus during egg laying, and that the enhanced activity of hypothalamic dopamine and pentraxin promotes the synthesis and release of Vasoactive Intestinal peptide (VIP) (Gumułka et al., 2020), which in turn promotes the secretion of PRL through feedback (Avital-Cohen et al., 2012).

Under natural conditions, most poultry (birds) are seasonal breeding animals, and breeding status is divided into breeding and non-breeding seasons due to changes in sunlight throughout the year. However, domesticated poultry, such as chickens and ducks, can breed throughout the year (Kuenzel et al., 2015; Mishra et al., 2017; Mishra et al., 2018). In both the production of breeders and laying poultry, light management is very important and directly affects laying performance (Chew et al., 2021; House et al., 2021). In practical production, the light duration during the egg-laying photoperiod is generally maintained at 16–18 h in non-seasonal breeding birds, and feed intake was limited every day (Cui et al., 2021; Ouyang et al., 2021). Female mountain ducks start laying eggs at 100-day-old of age, laying 280 to 300 eggs per year, no nesting characteristics (Cao et al., 2020). There are still no systematic studies reporting a light duration that what is the underlying regulatory mechanism in too long-day photoperiod or too short-day photoperiod for optimal follicle development and laying performance of non-seasonal breeding birds. Many scholars have reported that OPN5-mediated light regulation of avian reproduction mostly focuses on OPN5 regulation of GnRH secretion through the TSH pathway (Kuenzel et al., 2015; Mishra et al., 2017; Mishra et al., 2018). In the pathway of regulating avian reproductive activities by light, OPN5 and TSH, as two important mediators, are in the upstream and downstream between light and reproductive axis, respectively, and they show consistent changes in the regulation of avian reproductive activities by light (Zhu et al., 2019a; Zhu et al., 2019b). OPN5, as a photoreceptor, is upstream in the pathway of light-regulated reproductive activity and is the main factor mediating light changes (Nakane and Yoshimua, 2019). There is the evidence that the knockdown of OPN5 *via* small interfering RNA antisense in the MBH revealed that there is an inhibitory input in the photoinduced regulation of *TSHβ* mRNA expression (Stevenson and Ball, 2012). In the previous work we have carried out, some direct results on the association between OPN5 and TSH have been obtained but not published, which was that active immunization of ducks with OPN5 immunogen was found to promote OPN5 expression and upregulate TSHβ and DIO2 expression, which in turn regulate HPG axis-related genes to promote follicle development in mountain ducks. However, there are no reports on whether the TSH-DIO2/DIO3 pathway regulates follicle development through the production of key regulators of the avian reproductive axis including GnRH, GnIH, VIP, and PRL, and whether different light durations are associated with the expression of these key regulators. The existence of a dose-dependent relationship between different light durations and the expression of key regulators in these regulatory pathways has not been reported. What is the regulatory pathway difference between seasonal breeding avian species (including short-day breeding avian species and long-day breeding avian species) and non-seasonal breeding avian species? These questions will not only help to elucidate the mechanism of OPN5 in regulating breeding activities of birds but also provide important guidance for light management in the production of breeding and laying poultry. In our study, we examined the egg laying rate, feed return, ovarian performance, reproductive hormone levels, and reproductive gene and protein expressions of mountain ducks under different light treatments to understand the relationship between follicular development, OPN5 expression patterns, reproductive axis hormone secretion, and gene expression in ducks under different light regimes. Moreover, we revealed that the OPN5 regulating mechanism of follicular development occurs through the TSH-DIO2/DIO3 pathway.

## 2 Materials and Methods

### 2.1 Ethics Statement

All experimental procedures in this study were performed in accordance with the Regulations for the Administration of Affairs Concerning Experimental Animals (State Science and Technology Commission in China, 1988) and EU Directive 2010/63/EU for animal experiments. All animal experiments were approved by the Animal Care and Use Committee of Zhongkai University of Agriculture and Engineering (Guangzhou, China).

### 2.2 Animals and Experimental Design

**FIGURE 1:**
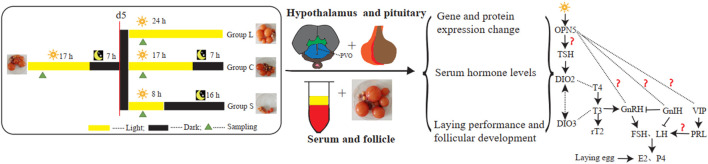
Experimental design All mountain ducks were randomly divided into three types of group. All samples were collected at d0, d5, d8, d20, d35. Serum samples in the three types of group were collected for analysis of serum hormone levels. Hypothalamic and pituitary tissue in the in the three types of group were collected for related genes and proteins analysis.

The trial was undertaken at the Institute of Animal Health, Guangdong Academy of Agriculture Sciences, Guangzhou, Guangdong Province, China. One hundred and twenty normal laying mountain ducks were pre-fed for 15 d in 150-day-old with limited food (150 g per duck each day) and unlimited water at three constant rooms temperature of 21°C and light intensity of 400 lux under a 17 h light/7 h dark cycle. At 165-day-old of age, all ducks were randomly divided into three groups, with 40 birds in each group kept in cages, among them two ducks in a cage (Figure 1). They were initially under 17 L:7D light conditions at light intensity of 400 lux for 5 days. Then, all ducks in the group receiving the long-day photoperiod of light (group L), the group receiving the 17 L:7D photoperiod of light (group C), and the group receiving the short-day photoperiod (group S), were maintained under 24 L:0D, 17 L:7D, and 8 L:16D light conditions for 30 days, respectively. All samples were collected at 2 h after lights-on zeitgeber time 6 (ZT6), which for group S birds was 6 h after dark onset, and for group C birds 15 h before dark onset, and for group L birds 24 h (lights-on was the same for group S and group C). The number of eggs laid in each group was recorded each day and blood samples were collected at d 0, 5, 8, 20, and 35 (*n* = 5 or *n* = 8), five samples per group for qPCR, five or eight for hormone assays (including five samples per group for d0 and d20, eight for d5, d8 and d35), five for western blots and three for immunohistochemistry, in which d 0, 5, 8, 20, and 35 were August 6, August 11, August 14, August 25, and 10 September 2020, respectively, China. The serum was separated and reserved for analysis of serum hormone levels, follicular development was examined, gonadal index was calculated, and hypothalamic and pituitary tissue samples were collected and reserved (−80°C) for tests of expression of related genes and proteins.

### 2.3 Detection of Serum Reproductive Hormones

The determination of E2/P4/GnRH/T/PRL/LH in serum was performed according to the kit instructions. These kits were: Duck Estradiol (E2) ELISA KIT (CUSABIO, Shanghai, China), Duck Progesterone (P4)/GnRH/Testosterone ELISA KIT (Elabscience, Shanghai, China), Duck Prolactin (PRL) ELISA KIT, and Chicken Luteinzing hormone (LH) ELISA KIT (SAB, Shanghai, China). Among them, the detection range of E2 is 40–1000 pg/ml, and the coefficient of variation CV is less than 10%; the detection range of P4 is 0.31–20 ng/ml, and the coefficient of variation CV is less than 10%; the detection range of GnRH is 15.63–1000 pg/ml, and the coefficient of variation CV is less than 10%; the detection range of T is 0.31–20 ng/ml, and the coefficient of variation CV is less than 10%; the detection range of PRL is 40–1000 *µ* IU/ml, coefficient of variation CV less than 8%; the detection range of LH is 0.5–100miu/ml, and the coefficient of variation CV is less than 8%.

### 2.4 Detection of Gene Expression Levels

Tissue RNA was extracted by trizol conventional method and cDNA was obtained by reverse transcription of RNA with PrimeScript^TM^RT reagent Kit with gDNA Eraser (TaKaRa, Tokyo, Japan). Real-time quantitative polymerase chain reaction (qRT-PCR) was used to quantify the expression of *OPN5*, *TSHβ*, *TRH*, *TSHR*, *THRB*, *DIO2*, *DIO3*, *GnRH*, *GnIH*, *PRL*, *GnRHR*, *GnIHR*, *FSH*, and *LH* mRNA expression levels in hypothalamic or pituitary tissue. The qRT-PCR analysis was performed using the Applied Biosystems Quant Studio seven Flex Real-Time PCR System (Thermo Fisher, United States). Based on the reference sequence on NCBI, the fluorescent quantitative primers for the above genes and the primers for the β-actin gene as an internal reference were designed using Primer 5.0 (Table 1) and synthesized by Sangon Biotech Co, Ltd., Using cDNA as a template, a 10 µL reaction system was prepared: PowerUPTM SYBR™ Green Master Mix (Thermo Fisher, United States) 5 μL, upstream and downstream primers 0.1 µL each, ddH2O 3.8 µL, cDNA template 1 µL. Reaction conditions were set: 50°C pre-denaturation for 2 min, 95°C for 10 min1 cycle; 95°C for 15 s, T_m_°C for 1 min, 40 cycles. Three replicates of each sample were normalized with β-actin as an internal reference gene using the 2^−ΔΔCT^ method.

**TABLE 1:** Primers for real-time fluorescent quantitative PCR

Gene	Primer Sequence (5´-3´)	Annealing temperature (°C)	Product size (bp)	GenBank No.
OPN5	F: ACC​AGG​ATC​CAG​AAC​AGC​CA	56	80	XM_019613613.2
	R: GCA​ATG​AGG​AAT​CCG​GCA​CA			
GnRH	F: CTG​GGA​CCC​TTG​CTG​TTT​TG	59	209	XM_013176792.1
	R: AGG​GGA​CTT​CCA​ACC​ATC​AC			
GnIH	F: AAA​GTG​CCA​AAT​TCA​GTT​GCT	58	128	XM_015853673.2
	R: GCT​CTC​TCC​AAA​AGC​TCT​TCC			
VIP	F: TCA​AAC​GCC​ACT​CTG​ATG​CT	60	124	XM_035538857.1
	R: GAG​GGG​TTT​AGC​TCT​TCC​TGG			
DIO2	F: GAC​GCC​TAC​AAG​CAG​GTC​AA	57	119	XM_013094234.4
	R: GTT​CCA​CAC​TTG​CCA​CCA​AC			
DIO3	F: AGA​TGC​TAC​TGA​TGC​CCA​CG	57	271	XM_013199473.1
	R: CCG​AAG​TTG​AGG​ATG​AGG​GG			
TRH	F: TGG​TGA​AGT​AAA​TTA​CCA​GAA​CAC	60	98	XM_013182272.1
	R: CCT​AAA​TGG​GGA​CAC​TCA​CTC​AC			
TSHβ	F: CGT​GTG​CAC​ATA​CAA​AGA​GAT	56	162	NM_001310425.1
	R: GCA​ATA​GTT​TGG​CCT​AAC​CTT			
THRβ	F: GCT​TAT​CTC​TGG​GCA​ATG​TGA​C	60	299	XM_038174583.1
	R: TTG​AAG​CGA​CAT​TCC​TGG​CA			
TSHR	F:CCCCAACATCTCTAGGATTGAA	60	200	XM_009646634.2
	R: CTG​AAG​TCA​TGA​AAG​GAT​TAT​CTG​C			
GnRHR	F: TCT​GCT​GGA​CCC​CCT​ACT​AC	62	127	NM_001012609.1
	R: TCC​AGG​CAG​GCA​TTG​AAG​AG			
GnIHR	F: CAT​CCT​GGT​GTG​CTT​CAT​CG	56	164	XM-005028365.3
	R: ACA​TGG​TGT​TGT​CAA​AGG​GC			
PRL	F: ACC​TCC​TTG​CCT​ATC​TGC​CC	60	180	NM_001310372.1
	R: TTG​TAA​TGA​AAC​CCC​GAC​CC			
FSH	F: GTG​GTG​CTC​AGG​ATA​CTG​CTT​CA	60	209	XM_031607398.1
	R: GTG​CAG​TTC​AGT​GCT​ATC​AGT​GTC​A			
LH	F: CCA​GGC​CTC​CTG​CAC​CTA​C	60	115	MK820637.1
	R: GGCGCAGCGGCAGCTCAG			
β-actin	F: CCT​CTT​CCA​GCC​ATC​TTT​CTT	60	110	XM_035563367.1
	R: TGT​TGG​CAT​ACA​GGT​CCT​TAC			

### 2.5 Protein Extraction and Western Blot Analysis

The total protein of the hypothalamus was extracted and total protein concentration was determined by BCA (Beyotime, Shanghai, China). After denaturation by SDS (Beyotime, Shanghai, China), the protein was sampled using sodium dodecyl sulfate-polyacrylamide gel electrophoresis (SDS-PAGE) of 10–15%, and electrophoresis was performed at 80 V for 15 min and at 120 V for 60 min. Upon completion of electrophoresis, the protein was transferred to polyvinylidene fluoride (PVDF) membranes at 200 mA and after blocking with phosphate-buffered saline with Tween 20 (PBST) containing 5% fat-free milk, PVDF membranes were co-incubated with the antibodies, anti-β-actin (1:5,000, Proteintech, Wuhan, China), anti-OPN5 (1:1000, self-made antibody), anti-THRA/THRB (C3) (1:1000, Invitrogen, United States), anti-GnRH-1-Ab (1:1000, Affinity Biosciences, OH, United States), anti-GnIH(1:1000, self-made antibody)and anti-DIO2 (1:1000, Affinity Biosciences, OH, United States). They were then incubated overnight at 4°C and washed five times at TBST each for 3 min. The secondary antibodies, goat anti-rabbit (1:10000, Affinity Biosciences, OH, United States) and goat anti-mouse (1:10000, Abcam, Cambridge, United Kingdom), were incubated for 1 h at room temperature and washed three times at TBST for 10 min each. Proteins were detected using the ECL kit (Beyotime, Shanghai, China), and visualized using a Tanon-5200Multi device (Tanon, Shanghai, China), photographed and stored. Densitometry analysis was performing using ImageJ software.

### 2.6 Detection of Immunohistochemistry

Deparaffinizing and rehydrating the paraffin section: the sections were placed into xylene I for 15 min, xylene II for 15 min, xylene III for 15 min, absolute ethanol I for 5 min, absolute ethanol II for 5 min, 85% alcohol for 5 min, 75% alcohol for 5 min, and then rinsed in distilled water. Antigen retrieval: The tissue sections were placed in a repair box filled with citric acid (pH 6.0) antigen retrieval buffer for antigen retrieval, within a microwave oven, heated on medium power for 8 min until boiling, kept warm for 8 min, and then heated again, this time on medium-low power, for 7 min. During this process, excessive evaporation of buffer should be prevented and the sections should not be allowed to dry. To cool the sections to room temperature before proceeding, the sections were placed in PBS (pH 7.4) and shaken on the decolorization shaker three times for 5 min each. Blocking endogenous peroxidase activity: the sections were placed in 3% hydrogen peroxide and incubated at room temperature in darkness for 25 min. The sections were placed in PBS (pH 7.4) and shaken on a decolorizing shaper three times for 5 min each. Serum sealing: 3% BSA was added to the circle to evenly cover the tissue, and the tissues were sealed for 30 min at room temperature (the primary antibody was sealed with normal rabbit serum from a goat source while other sources are sealed with BSA). Primary antibody incubation: the sealing solution was gently removed, the primary antibody prepared with PBS (pH 7.4) and then a known proportion was added to the sections and the sections placed flat in a wet box to be incubated overnight at 4°C (a small amount of water was added to the wet box to prevent evaporation of antibodies). Secondary antibody incubation: the sections were placed in PBS (pH 7.4) and washed by shaking on the decolorizing shaker three times for 5 min each. After the sections were slightly dried, the tissues were covered with a secondary antibody (HRP labeled) from the corresponding species of primary antibody and incubated at room temperature for 50 min. DAB chromogenic reaction: the sections were placed in PBS (pH 7.4) and shaken on the decoloring shaker three times for 5 min each. DAB color developing solution, newly prepared, was added to the circle after the sections had slightly dried. The color developing time was controlled under the microscope, until the optimal color of brownish yellow was reached. Subsequently, the sections were rinsed with tap water to stop the reaction continuing. Nucleus counterstaining: the sections were counterstained with hematoxylin stain solution for about 3 min, washed with tap water, differentiated with hematoxylin differentiation solution for several seconds, then washed again with tap water, treated with hematoxylin returning blue solution; and washed once more with tap water. Dehydration and mounting: The sections were placed in 75% alcohol for 5 min, then 85% alcohol for 5 min, absolute ethanolⅠfor 5 min, anhydrous ethanol Ⅱfor 5 min, n-butanol for 5 min, and then xyleneⅠfor 5 min. Now dehydrated and transparent, they were then removed from the xylene and allowed to slightly dry, and then mounted with neutral gum. Staining of tissue was visualized using 3D Histech Quant Center 2.1 (3D Histech, Hungary).

The nucleus of hematoxylin stained is blue, and the positive expression of DAB is brownish yellow.

### 2.7 Statistical Analysis

Statistical analyses were performed using Prism 7 (Graphpad Sofware Inc., La Jolla, CA, United States). Multiple comparison analysis was performed using a two-way ANOVA followed by Tukey’s post-hoc correction for multiple comparisons. All experimental data were analyzed using the means or the means ± standard error of mean (S.E.M.). and differences were considered to be significant at *p* < 0.05. Graphics were plotted using the ggplot2 package in R.

## 3 Results

### 3.1 Effects of Different Photoperiods on Laying Performance and Follicular Development

**FIGURE 2:**
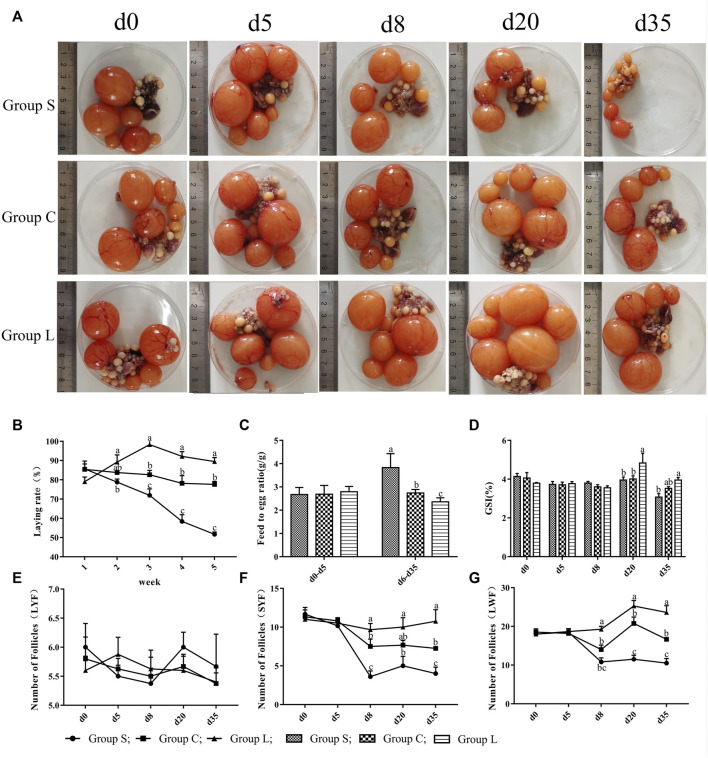
Laying performance and follicular development of mountain ducks under different photoperiods. 1**(A)**, pictures of the follicles of the mountain ducks; 1**(B)**, egg production of mountain ducks; 1**(C)**, feed-to-egg ratio of mountain ducks; 1**(D)**, ovarian index of mountain ducks; 1**(E)**,1**(F)**,1**(G)**, number of follicles of mountain ducks. Consecutive letters **(A,B)** indicate significant differences (*p* < 0.05), and discrete letters **(A,C)** indicate highly significant differences (*p* < 0.01), the same as below.

The results showed no difference in laying rate, feed-to-egg ratio, and ovarian index in the three groups under the same light conditions (17 L:7D). After experiencing different light conditions, the average cumulative number of eggs laid per duck was 26.0, 22.0, and 19.6 in group L, C, and S, respectively. Prolonged light exposure increased laying performance and reduced light exposure suppressed laying performance, with the highest laying performance exhibited by group L (Figure 2B). The trend of feed-to-egg ratio (Figure 2C) was opposite to laying performance, with the highest feed-to-egg ratio in group S, which was significantly higher than group C and group L (*p* < 0.05), while group C was significantly higher than group L (*p* < 0.05). Ovarian development results (Figures 2D–G) showed that longer light exposure significantly increased the follicular ovarian index (*p* < 0.05) and shortened light exposure significantly decreased the ovarian index (*p* < 0.05). There was no significant difference in the number of LYF (Large Yellow Follicle) in the three groups under different photoperiods. However, the number of SYF (Small Yellow Follicle) and LWF (Large White Follicle) was highest in group L and significantly higher (*p* < 0.05) at d 8, d 20, and d 35 than in group C, and significantly higher (*p* < 0.05) in group C than in group S.

### 3.2 Serum Hormone Levels of the HPG Axis

**FIGURE 3:**
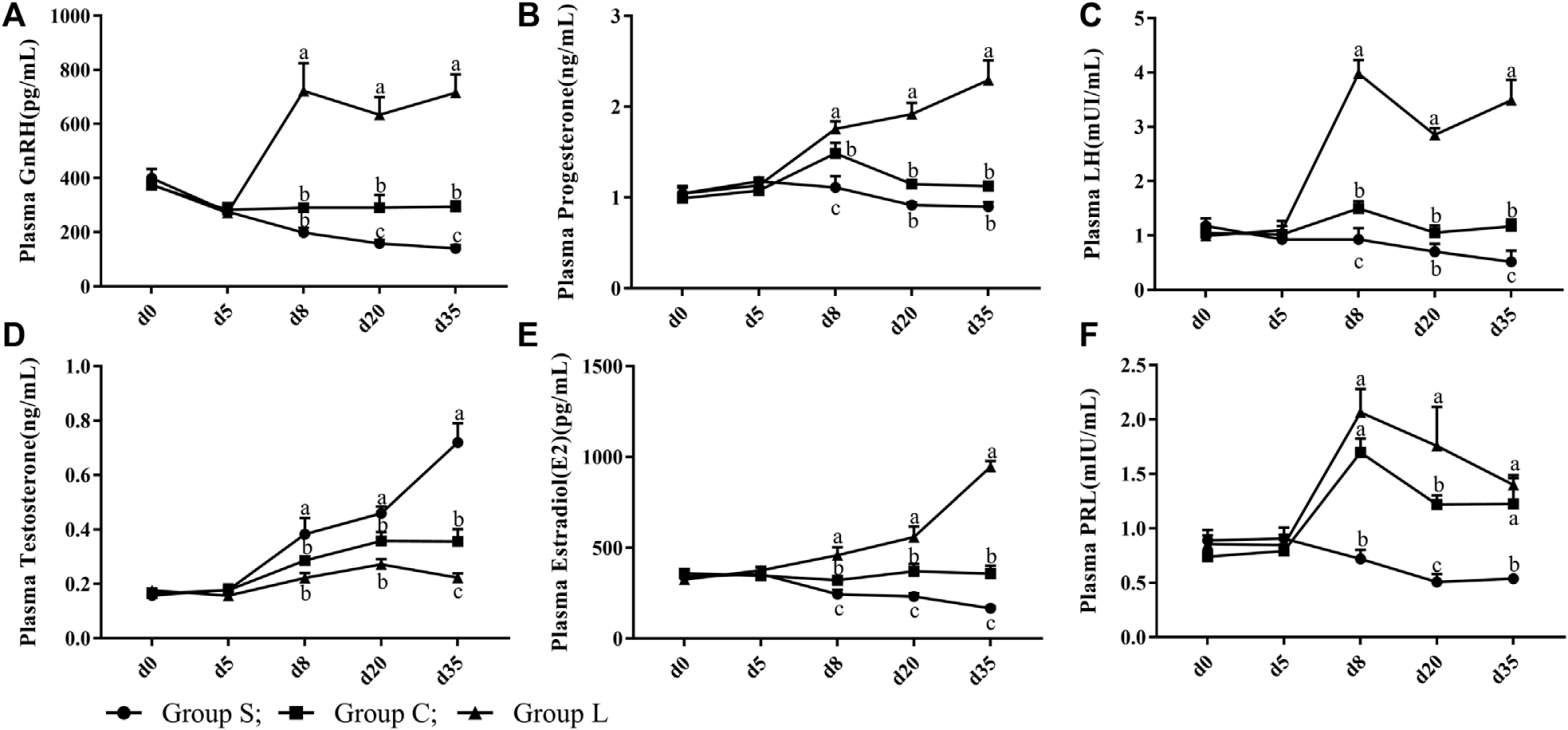
Serum reproductive hormone levels of mountain ducks under different photoperiods. **(A– F)** indicated the levels of GnRH, Progesterone, LH, Testosterone, Estradiol, PRL reproductive hormones, respectively.

Duck serum hormones showed no significant differences (*p* > 0.05) in the GnRH, P4, LH, T, PRL, and E2 levels in serum under the same light condition (17 L:7D; Figures 3A–F). After treatment with different light conditions (Figures 3A–F), the levels of GnRH, P4, LH, and E2 in serum were significantly increased (*p* < 0.05) and the level of testosterone was significantly decreased (*p* < 0.05) as the duration of light exposure increased. In contrast, the levels of GnRH, P4, LH, E2, and PRL in serum were highly significantly inhibited (*p* < 0.05), while levels of testosterone were highly significantly increased (*p* < 0.05) after the short-day photoperiod treatment.

### 3.3 OPN5 Expression Levels Under Different Photoperiods

**FIGURE 4:**
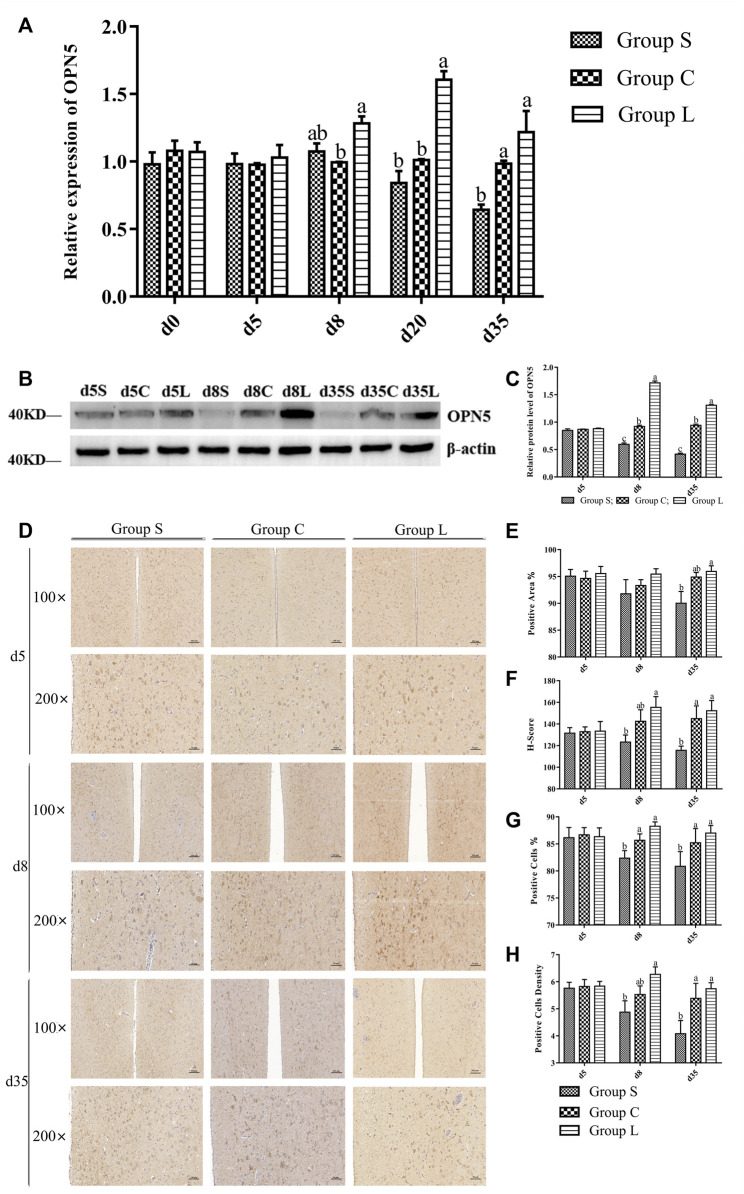
OPN5 expression levels of protein and gene of mountain ducks under different photoperiods. **(A)**, the gene expression of OPN5; **(B)**, Western Blot results of relative protein levels of OPN5; **(C)**, changes in the protein expression of OPN5; **(D)**, the immunohistochemical staining images of OPN5; **(E–H)** were quantitative analysis of the immunohistochemical results of OPN5, indicating the analysis results of positive area, H-Score, positive cells, and positive cells density, respectively

Detection of the expression pattern of hypothalamic OPN5 revealed (Figures 4A–C) that OPN5 in mRNA and protein expression were both at stable levels under 17-h stable light conditions. Both gene and protein expression of OPN5 changed when light exposure was changed. OPN5 mRNA expression in group L was significantly higher than that in group C at d eight and d 20 (*p* < 0.05), and d 35 expression was reduced compared with d eight and d 20, but still higher than the control level (*p* > 0.05). These results were consistent with the results of western blots (Figures 4B,C) and immunohistochemistry (Figures 4D–H). The expression of both the OPN5 gene and protein decreased gradually in group S after the short-day photoperiod treatment and was significantly lower than that in group C at d 35 (*p* < 0.05).

### 3.4 Expression Levels of Genes and Proteins in the TSH-DIO2/DIO3 Pathway

**FIGURE 5:**
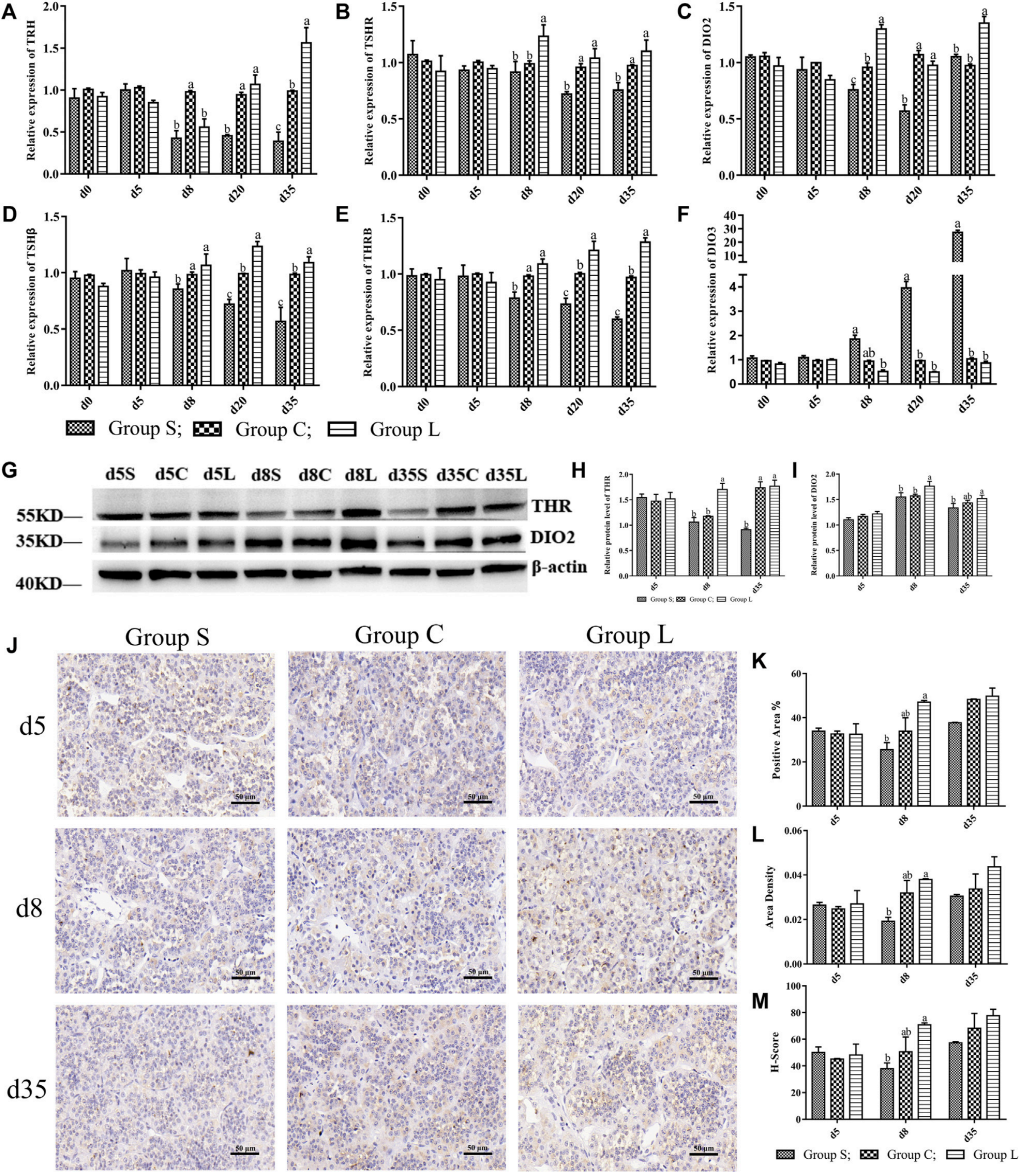
Expression levels of genes and proteins in TSH-DIO2/DIO3 pathway under different photoperiods Expression of genes and proteins related to the TSH-DIO2/DIO3 pathway. **(A–F)** indicated the gene expression levels of TRH, TSHR, DIO2, TSHβ, THRβ, and DIO3, respectively; **(G)**, Western Blot results of relative protein levels of THR and DIO2; **(H)** and **(I)**, changes in the protein expression of THR and DIO2; **(J)**, the results of immunohistochemical staining images of TSH (400 × ); **(K–M)** were the results of TSH immunohistochemical quantification, indicating the results of positive area, Area density, H-Score, respectively.

Detection of genes in the TSH-DIO2/DIO3 pathway revealed that the gene expression patterns of *TRH*, *TSHβ*, *TSHR*, *DIO2*, and *THRβ* in the pathway were basically consistent with the expression pattern of *OPN5*, which gradually increased under long-day photoperiod and decreased under short-day photoperiod. While the expression pattern of *DI O 3* was inversely correlated with *OPN5*, *DIO3* in group S was significantly higher than was *DIO3* in group C (*p* < 0.05) (Figures 5A–F). Detection of TSH-DIO2/DIO3 pathway proteins revealed that the results of THR and DIO2 western blots (Figures 5G–I) and the immunohistochemical results of TSHβ (Figure 5J–M) demonstrated an increasing trend under long-day photoperiod and a down-regulation of TSH, THR, and DIO2 protein expression under short-day photoperiod, which were positively correlated with the trend of OPN5 protein expression.

### 3.5 Expression Levels of the Main Factors of the HPG Axis

**FIGURE 6:**
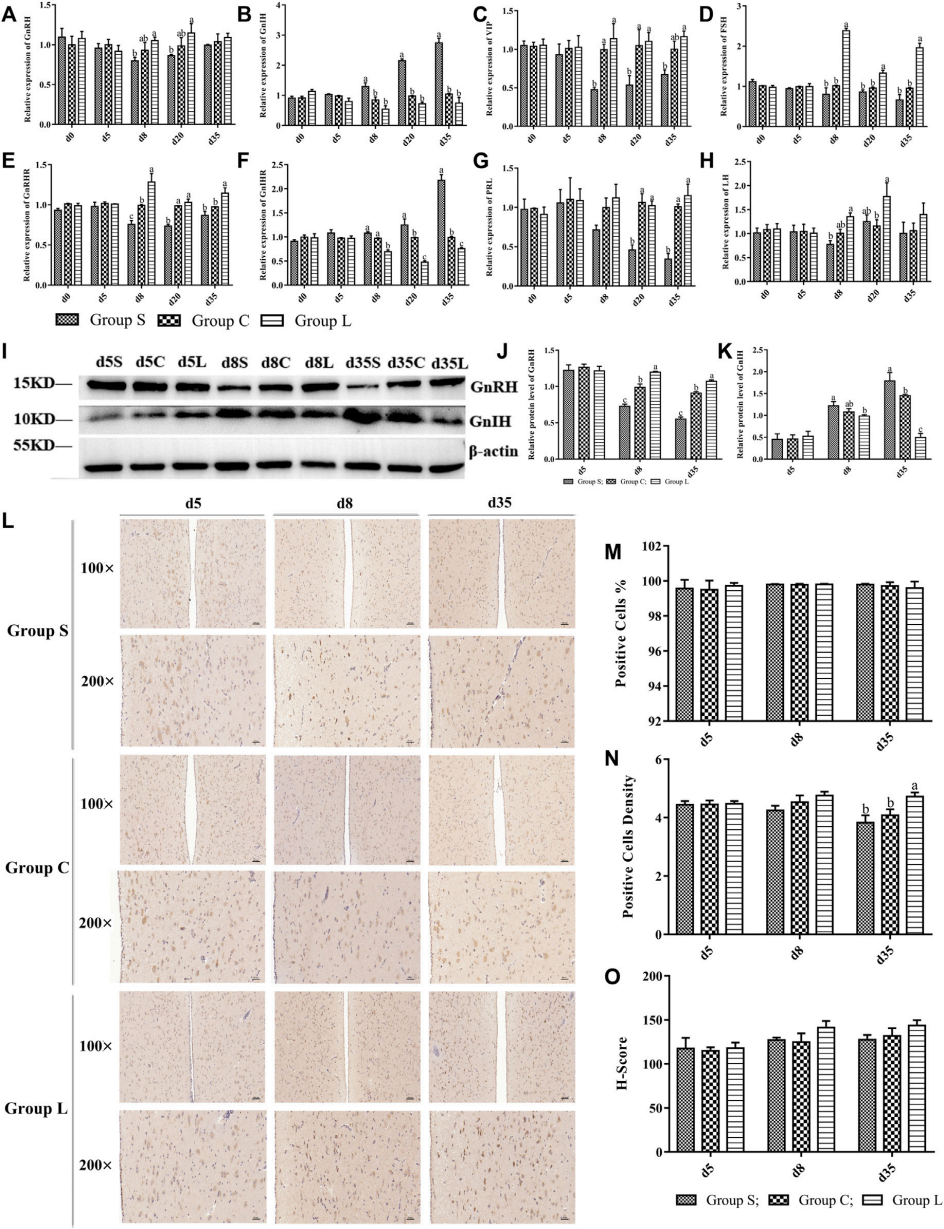
Expression levels of main factors of HPG axis in mountain ducks under different photoperiods. Expression of HPG axis-related genes and proteins. **(A–H)** indicated the expression levels of the genes of GnRH、GnIH、VIP、FSH、GnRHR、GnIHR、PRL and LH, respectively; **(I)**, Western Blot results of relative protein levels of GnRH and GnIH; **(J)** and **(K)**, changes in the protein expression of GnRH and GnIH; **(L)**, the immunohistochemical staining images of GnRH; **(M–O)** were quantitative analyses of the immunohistochemical results of GnRH, indicating positive cells, positive cells density, and H-Score, respectively.

The results of the expression of genes related to the HPG axis found that the relative gene expression of *GnRH*, *GnRHR*, *FSH*, *LH*, *VIP,* and *PRL* gradually increased in group L, and the relative gene expression of *GnIH* and *GnIHR* gradually decreased; the opposite of group S (Figures 6A–H). This was consistent with the reproductive status of the ducks. Moreover, the gene and protein expression patterns of GnRH were consistent with those of OPN5 and THRβ, and the gene and protein expression patterns of GnIH were opposite to that of OPN5, GnRH, and THRβ. Therefore, this study suggests that OPN5 may affect follicle development by regulating the TSH-DIO2/DIO3 pathway in order to impact the production of key regulators of the avian reproductive axis, including GnRH, GnIH, VIP, and PRL. (Figure 6).

### 3.6 Expression Correlation Analysis Between Positive and Negative Regulatory Factors

**FIGURE 7:**
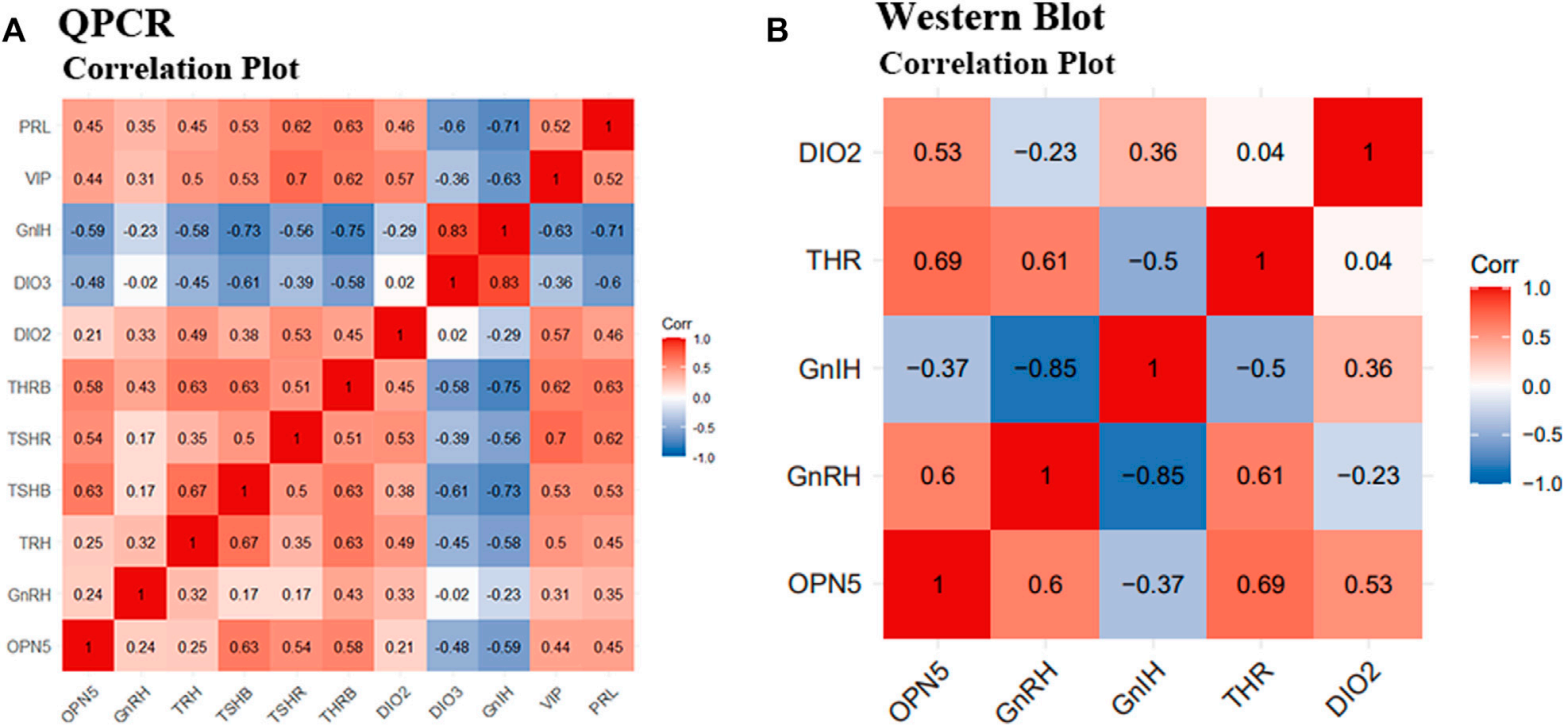
Correlation analysis between positive and negative regulatory factors of reproduction activities under different photoperiods. Correlation analysis of QPCR results and Western Blot results. **(A)** indicates the correlation analysis of the gene results of OPN5, GnRH, TRH, TSHβ, TSHR, THRβ, DIO2, DIO3, GnIH, VIP, and PRL, respectively; **(B)** indicates the correlation analysis of the protein results of OPN5, GnRH, GnIH, THR, and DIO2, respectively.

At the gene level, OPN5 was positively correlated with GnRH, TRH, TSHβ, THRβ, DIO2, VIP, and PRL, and was negatively correlated with DIO3 and GnIH. The results of QPCR showed that the top seven most positively correlated gene pairs were, in ascending order: OPN5 and TSHβ (r = 0.63), TSHβ and PRL (r = 0.63), TRH and THRβ (r = 0.63), TSHβ and THRβ (r = 0.63), TSHβ and TRH (r = 0.67), VIP and TSHR (r = 0.7), and GnIH and DIO3 (r = 0.83). The top three most negatively correlated pairs of genes were TRH and PRL (r = -0.75), GnIH and THRβ (r = -0.75), and GnIH and TSHβ (r = -0.73). (Figure 7A).

At the protein level, OPN5 was positively correlated with GnRH, THR, DIO2, and was negatively correlated with GnIH. Western blot results showed that the three groups of proteins with the highest correlation were GnRH and GnIH (r = -0.85), OPN5 and THR (r = 0.69), and GnRH and THR (r = 0.61). (Figure 7B).

## 4 Discussion

OPN5 is a deep brain photoreceptor that mediates the regulation of light on avian reproductive activity (Kang and Kuenzel, 2015; Beaudry et al., 2017). To date, studies on OPN5 have mainly focused on seasonally breeding birds, and a large number of studies have shown that their light perception can regulate the secretion of TSHβ through the mediation of OPN5, which in turn feeds back to the HPG axis to cause the release or inhibition of the corresponding hormones, thus participating in the regulation of gonadal development and the reproductive regulation process of animals (Zhu et al., 2019a; Zhu et al., 2019b). It is unclear that whether OPN5 regulating follicular development through TSH-DIO2/DIO3 system responds to different photoperiods in non-seasonal laying ducks.

In the present study, we observed that the morphology and function of the three groups of follicles were dramatically changed by altering the photoperiod. Compared to 17 h light time, 24 h light time showed higher egg production and lower egg ratio, while 8 h light time suppressed egg production and increased egg ratio in the mountain ducks. GnRH, P4, E2, LH and PRL levels in serum increased with longer light time, which increased the amount of LYF, SYF and LWF. Our results confirmed previous observations that male Japanese quail transferred to different photoperiods undergo a rapid change in plasma reproductive hormones and gonad weight (Oishi and Konishi, 1978; Wada, 1993; Henare et al., 2011). The results of the study showed that plasma levels of GnRH, E2, P4, LH, PRL and T dramatically changed by altering the photoperiod after 3 d. Compared to the 17 h photoperiod, longer light time promoted follicular development and plasma levels of GnRH, E2, P4, LH and PRL hormone in mountain ducks were significantly increased in the 24 h photoperiod, whereas the 8 h photoperiod inhibited follicular development and promoted plasma levels of testosterone hormone in mountain ducks. Studies have shown that longer light exposure elevates the gene level of GnRH (Zhu et al., 2017), while GnRH promotes the secretion of FSH and LH, and then LH promotes secretion levels of P4 and E2, which ultimately induce gonadal development and enhance reproductive activity (Hanlon et al., 2021). In quail, transfer from short-day photoperiod to long-day photoperiod causes a significant increase in serum LH, FSH and other reproductive hormone levels and gonad weight (Henare et al., 2011). Meanwhile, E2 and P4 are the main indicators of reproduction (Porcu et al., 2018), and LH and PRL are also potential key indicators of reproduction in poultry (Etches RJ Gordon Memorial Lecture, 1998; Tan et al., 2021a; Geng et al., 2022). Elevated plasma levels of PRL and LH in breeding ducks can promote egg production (Cui et al., 2021).

The link between OPN5 and TSH is very strongly in the hypothalamus and pituitary gland (Nakane and Yoshimura, 2014; Kuenzel et al., 2015; Mishra et al., 2017). In terms of the experiment, we investigated the relationship between OPN5 and TSH under different photoperiods. We observed that prolonged light time elevated the expression levels of OPN5 on proteins and genes, and there was a significant positive correlation between OPN5 and TSH under different photoperiods, which was inextricably linked to the expression of reproduction-related genes. In seasonally breeding avian species, seasonal changes in reproductive activity are mainly controlled by light, and different light durations or wavelengths may lead to an increase or decrease in OPN5 expression, which in turn regulates the animal’s reproductive activity. Prolonging the light time promotes the expression of OPN5 and TSHβ, enhancing egg production in the Hungarian white geese as well (Kuenzel et al., 2015). The expression of OPN5 is higher under white and red light conditions than under blue and green light conditions, and enhancing the egg production performance in the Yangzhou geese (Zhu et al., 2019b). *OPN5* mRNA expression and testicular development are promoted by prolonging light time in the *Gallus* (Kang and Kuenzel, 2015). In quail, it is found that reducing OPN5 expression significantly suppressed TSHβ expression (Nakane et al., 2014). The results of the present study suggested that OPN5 played an important role in the regulation of avian reproductive activity by mediating photoperiod in non-seasonal breeding avian. OPN5 affects TSH, which activates the DIO2/DIO3 conversion system (Ono et al., 2008), which in turn affects the GnRH/GnIH system and the GnRHR/GnIHR system, altering the expression of related reproductive genes and causing changes in the corresponding reproductive hormones, thus regulating the ovarian functional system (Banerjee and Chaturvedi, 2018). Furthermore, OPN5 has been shown to mediate light signaling and cause a drift in rhythmic phase, which in turn is involved in the regulation of animal biological rhythms and reproductive functions, such as the association of altered sexual behavior and GnRH release (Prasad et al., 2015; Han et al., 2017). Non-seasonal reproductive activity can be driven by light stimulation and is dependent on neuroendocrine regulation, whereby hormones in the HPG axis are altered to initiate and maintain gonadal development (Mauro et al., 1989; El Halawani and Rozenboim, 1993; Dunn et al., 2004; Johnson, 2015). In birds, the activity of the HPG axis is strictly controlled by the level of GnRH. GnRH neurons in the hypothalamus have dynamic morphological plasticity in response to photoperiodic changes that regulate the seasonal secretion of GnRH in the hypothalamus (Jansen et al., 2003; Yamamura et al., 2004; Lehman et al., 2010). Whereas TSH is thought to be a key signaling molecule regulating reproductive seasonality in birds (Nicholls et al., 1988; Yoshimura et al., 2003; Nakao et al., 2008), TSH changes markedly from the nonreproductive active phase to the active phase (Donham, 1979) and T3 controls the seasonal pulse of GnRH release (Hanon et al., 2008; Nakane and Yoshimura, 20102008). The reproductive activity of the HPG axis with the release of GnRH/GnIH and the negative feedback of gonadal reproductive hormones are topical studies (Kriegsfeld et al., 2015).

We found that prolonging light time elevated OPN5 expression levels in genes and proteins, while shortening light time down-regulated OPN5 expression levels in genes and proteins. The expression patterns of *GnRH*, *TRH*, *TSHβ*, *TSHR*, *DIO2*, *THRB*, *VIP* and *PRL* mRNA showed positive correlation with *OPN5* mRNA expression, while the expression patterns of *GnIH* mRNA was inversely correlated with *OPN5* mRNA expression by prolonging light time in mountain ducks. The protein expression of GnRH、TSHβ、DIO2 and THR was positively correlated with that of OPN5, while the protein expression of GnIH was inversely correlated with that of OPN5. These results suggested that OPN5 might affect the reproductive activity of the mountain ducks by modulating theTSH-DIO2/DIO3 pathway and thus the HPG axis. It has been reported that exposing *Gallus* to long-day photoperiod conditions for 3 days results in a significant increase in the number of cells positive for OPN5 in the brain, which is induced to promote *FSHβ* And *TSHβ* mRNA expression, thereby promoting the function of the reproductive system (Kang and Kuenzel, 2015). In quail, knockdown of siRNA-OPN5 can significantly inhibit *TSHβ* mRNA expression in long-day photoperiod (Nakane et al., 2014). In sparrows, prolonged light stimulates the synthesis of TSHβ and promotes the expression of *DIO2* mRNA, and it promotes the secretion of GnRH-I (Anand and Dixit, 2018). In Yangzhou geese, *OPN5* mRNA is upregulated by prolonged light, while the expression of OPN5 and TSH and DIO2 are positively correlated (Zhu et al., 2019b). In Hungarian white geese, prolonged light exposure increases the *OPN5* mRNA expression and *TSHβ* mRNA expression, and contributes to the upregulation of *VIP* and *PRL* gene expression in hypothalamic and pituitary tissues (Zhu et al., 2019a). The results of the present study were consistent with the above results. Meanwhile, network interactions between OPN5 and TSH, GnRH, GnIH, VIP and PRL regulate the reproductive activity of laying ducks.

In addition, PRL plays an important regulatory role in avian reproduction. In the present study, PRL was also significantly increased with long-day photoperiod treatment to promote reproductive performance of the mountain ducks, while under short-day photoperiod, PRL and VIP were significantly decreased, and follicular development and egg laying performance of the mountain ducks were also significantly reduced. Numerous studies have shown that high levels of PRL are key for the development and maintenance of bird nests, and that high levels of PRL could inhibit the secretion of the gonadotropic hormones of GnRH, FSH, and LH in the reproductive axis (Takeshi, 2017). However, it has also been shown that moderate PRL is necessary for follicle development in birds and that too low PRL under short-day photoperiod could inhibit follicle development and laying performance in birds (Li et al., 2020; Tan et al., 2021a; Tan et al., 2021b). Moderately long light exposure will preserve better egg laying performance in breeding birds (Chew et al., 2021; Cui et al., 2021), and reduction of endogenous PRL levels by immunization with PRL and VIP inhibits avian follicle development. When using long-day photoperiod exposure to promote Magang geese from the breeding stage to the resting stage, long-day photoperiod treatment at the early stage could promote PRL secretion while at the same time also promote follicle development and egg laying performance (Huang et al., 2008). These studies suggest that light, along with PRL, are important factors in the regulation of avian reproductive activity. This is consistent with the results of related studies, but why did high levels of PRL not inhibit follicle development under 24-h light conditions? Is it because PRL levels are not high enough to inhibit follicle development in mountain ducks? Does it correlate with the fact that nesting is no longer present in mountain ducks due to their artificial selection? These questions deserve further study. At the same time, what is the relationship between PRL—an important regulator involved in the regulation of follicle development and egg production performance ultimately influenced by light exposure—and the OPN5 and TSH-DIO2/DIO3 pathways in mountain ducks? Again, further studies are needed.

## 5 Conclusion

Long light exposure (17–24 h) promoted the follicular development in the ovary of mountain ducks and improved egg-laying performance. Long-day photoperiod might have affected the expression of OPN5, TSH, DIO2, VIP and PRL, decreased the expression of DIO3, and promoted follicle development. While short-day photoperiod down-regulated the expression of OPN5, TSH, DIO2, VIP and PRL, elevated the expression of DIO3 and inhibited follicle development and egg production performance. The results suggested that OPN5 might affect the expression and secretion of reproduction-related genes in the gonadal axis through the TSH-DIO2/DIO3 pathway under different photoperiods, which in turn regulated follicular development and egg-laying performance in mountain ducks.

## Data Availability

The original contributions presented in the study are included in the article/Supplementary Material, further inquiries can be directed to the corresponding authors.
